# Atypical Hemolytic Uremic Syndrome: A Case Report

**DOI:** 10.7759/cureus.4634

**Published:** 2019-05-10

**Authors:** Sohaib K Mohammed, Ateeq Mubarik, Bilal Nadeem, Khudadad Khan, Salman Muddassir

**Affiliations:** 1 Internal Medicine, Deccan College of Medical Sciences, Hyderabad, IND; 2 Internal Medicine, Oak Hill Hospital, Brooksville, USA; 3 Hematology and Oncology, KentuckyOne Health, Louisville, USA

**Keywords:** atypical hemolytic uremic syndrome (ahus), thrombocytopenia, hemolytic anemia.

## Abstract

Hemoytic uremic syndrome (HUS) is a rare type of thrombotic microangiopathies. Manifestations include thrombocytopenia, microangiopathic hemolytic anemia, and thrombi in small blood vessels. The prognosis is poor. Herein, we present a case of atypical HUS, which is very rare.

## Introduction

Hemolytic uremic syndrome (HUS) is a rare condition among the thrombotic microangiopathies (TMAs). Common manifestations of TMAs include thrombocytopenia, microangiopathic hemolytic anemia, and thrombi in small blood vessels, resulting in end-organ damage. The most common TMAs are Shiga toxin-producing *Escherichia coli* (*E. coli*) infection (STEC-HUS)-associated HUS and thrombotic thrombocytopenic purpura (TTP), followed by atypical HUS (aHUS) and secondary HUS due to the co-existing disease [1].

## Case presentation

A 37-year-old female with recurrent *E-coli* urinary tract infections was hospitalized with fever, vomiting, abdominal pain, lethargy, and altered mental status. She has a history of type I diabetes mellitus with retinopathy, gastropathy, and peripheral neuropathy and also had pancreatic and renal transplantation. Vital signs were within normal limits except for a temperature of 101.5 °F and respiratory rate of 24. Physical examination was unremarkable, but she was lethargic, though responding to a painful stimulus. Laboratory results revealed hemoglobin (Hgb) of 10.7gm/dl, platelets at 125,000/μl, a creatinine of 0.5, and peripheral blood smear revealed occasional schistocytes with reduced platelets on the day of admission. Computed tomography scan of the abdomen/pelvis revealed diffuse bladder wall thickening, with free fluid, and an edematous left transplanted kidney. Urinalysis showed evidence of a yeast infection. These findings were consistent with acute cystitis and pyelonephritis. A fecal impaction was also noted on imaging.

Despite being treated symptomatically and receiving an enema, the patient continued to experience intractable nausea and vomit. A nasogastric tube was placed because of a possible bowel obstruction. Ceftriaxone and fluconazole were prescribed for suspected pyelonephritis with sepsis. Hgb dropped from 11.3 on day one to 8.3 on day three (due to hemolysis), white blood cell count of 18,000 and platelets were at 32,0000/μl on day 2. An acute kidney injury was confirmed with fractional excretion of sodium at 2.7%, and the creatinine increased from 2.5 on day two, to 6.6 on day five, and to 8.17 on day seven. Complement studies revealed a C3 level of 57 (normal range: 80 to 160 mg/dL) and a C4 level of 16.4 (normal range: 16 to 48 mg/dL). Cytomegalovirus and Ebstein-Barr virus studies were positive. Haptoglobin level was 79.30; the lactic acid dehydrogenase was elevated at 584. ADAMTS 13 (von Willebrand factor-cleaving protease) was positive at 58%, which indicated aHUS. The international normalized ratio was 1.74. Iron studies were: iron 179, total iron binding capacity 213, and transferrin 152. Kidney biopsy was done to know the exact cause of rising creatinine levels without any obvious cause, which revealed a rare glomerulus with marked capillary congestion and intraluminal thrombus, due to thrombotic angiopathy. Immunofluorescence studies depicted linear and mesangial immunofluorescence for immunoglobulin G and fibrinogen, consistent with TMA. Based on these findings, classical atypical HUS was diagnosed. Platelets and eculizumab were infused on the third day. The patient's clinical condition improved, and the laboratory values stabilized; she has prescribed eculizumab upon discharge.

## Discussion

A case of atypical HUS is discussed in the vignette. Atypical HUS classically presents with the triad of thrombocytopenia, microangiopathic hemolytic anemia, and acute renal failure [2]. This patient presented in this manner with evidence for dysregulation of the complement system and had positive ADAMTS 13.

Atypical HUS is a multigenic complement-mediated disorder [2]. It is frequently associated with a genetic or acquired defect, yielding host cell dysregulation of complement. In many patients, a gastrointestinal or a urogenital infection precedes the clinical triad and leads to aHUS [3]. Mutations in the complement factor H (CFH) gene that encodes regulatory proteins are common genetic abnormalities in complement-mediated HUS [4-5]. Mutations in thrombomodulin, complement regulation, and coagulation protein are associated with aHUS [6-7]. Mutations of plasminogen, diacylglycerol kinase ε, and factor XII are also reported but linked directly to the complement system [8-11].

This condition is most often most commonly inflicts females and children. It can present with a wide myriad of symptoms however commonly presents with the classic triad. Some cases present with renal dysfunction and an abnormal creatinine, proteinuria, and hypertension; however, without thrombocytopenia or anemia. The most common extra-renal manifestation involves the central nervous system with obtundation, cerebrovascular accidents, seizures, or coma. This form has the worse prognosis in patients with HUS and end-stage renal disease (ESRD), requiring dialysis [1]. Since the advent of the anti-complementary therapy eculizumab, a monoclonal antibody to complement, the development of ESRD has been diminishing [3,5].

Complement-mediated HUS is diagnosed clinically by the demonstration of complement dysregulation by antibodies against complement factors or mutations in complement protein genes. Genetic screening should include CD46, C3, THBD, CHFR1, CHFR5, CFH, CFI, CFB, and DGKE [12]. To diagnose TMA, utilize laboratory evidence of hemolysis, thrombocytopenia, and renal failure. Once aHUS diagnosis is established, further studies aim to discover the etiologic pathology. Alternative differential diagnoses like TTP should be considered and ruled out. ADAMTS13 levels can aid in ruling out TTP.

## Conclusions

Atypical HUS has a poor prognosis eventually leading many patients to require dialysis. The mortality rate is high because the condition is often detected late in its course. Depending on the disease stage, treatments include plasma exchange, eculizumab, and/or dialysis. Plasma exchange therapy is the most frequent intervention. Some individuals who fail to respond to plasma therapy may require renal transplantation.
